# ‘Internet is easy if you know how to use it’: Doing online research with people with learning disabilities during the COVID‐19 pandemic

**DOI:** 10.1111/bld.12495

**Published:** 2022-08-01

**Authors:** Magdalena Mikulak, Sara Ryan, Siabhainn Russell, Sue Caton, Richard Keagan‐Bull, Rebecca Spalding, Francesca Ribenfors, Christopher Hatton

**Affiliations:** ^1^ Department of Social Care and Social Work Manchester Metropolitan University, Brooks Building Manchester UK; ^2^ Learning Disability England Birmingham UK; ^3^ Kingston University London UK; ^4^ Faculty of Health, Social Care and Education St George's University of London London UK

**Keywords:** empowerment issues, learning (intellectual) disabilities, research

## Abstract

**Background:**

The coronavirus disease 2019 pandemic changed the way we live, work, interact and do research. Many activities moved online, and digital inclusion became an urgent issue for researchers working with people with learning disabilities and other groups at risk of exclusion. This has generated new questions about how we conduct research and what it means to go into ‘the field’.

**Methods:**

We discuss our experience working across four qualitative research projects involving 867 participants with learning disabilities, conducted during the coronavirus disease 2019 pandemic.

**Findings:**

Moving research online resulted in often‐swift adaptations to research designs and practice, bringing new insights and benefits to our studies. The changing circumstances fostered innovation and greater flexibility and contributed to research becoming more accessible to many. However, doing research online also posed new challenges as well as amplified existing ones.

**Conclusions:**

The pandemic has made it easier for some people with learning disabilities to participate in research, but more needs to be done to improve the reach and quality of that participation. Researchers should make the process of participation as accessible as possible. It is also their job to question and challenge the conditions that create barriers to participation in research and to look for ways to change these. We make some recommendations on how this can be achieved.

## Accessible summary

- During the coronavirus disease 2019 (COVID‐19) pandemic, a lot of activities including research moved online. Access to the internet became even more important for people to be able to take part in the research.
- People with learning disabilities do not always have internet access or skills and support to use the internet. Devices and internet access can also be expensive. This means people with learning disabilities can be excluded from online activities and from research.
- In this article, we talk about four research projects with people with learning disabilities which we did during COVID‐19. We talk about what we did and about the changes we made to the projects because of the pandemic. We also talk about the things that worked well and the things that were difficult when we did research online.
- We think research should be accessible and we share some advice about how researchers can make it easier for people with learning disabilities to take part in research at a time when a lot of research happens online.

## 1 INTRODUCTION

In 2018, 10% of the adult UK population was described as ‘internet non‐user’, a number that has halved since 2011 (Office for National Statistics, 2019). The COVID‐19 pandemic intensified our online lives (Hantrais et al., 2021). Simultaneously the rapid move to working, learning, socialising, accessing healthcare and support online raised further awareness of the digital exclusion of certain groups including older adults, migrants, disadvantaged children and people with severe mental health illnesses (Coleman, 2021; Knights et al., 2021; Peckham & Spanakis, 2020; Ramsetty & Adams, 2020; Seifert, 2020). Similarly, people with learning disabilities continue to experience greater digital exclusion than nondisabled people (Alfredsson Ågren et al., 2020a; Caton & Chapman, 2016; Martin et al., 2021) even if this gap is slowly narrowing (D. D. Chadwick et al., 2019). In this article, we discuss how the pandemic affected research with people with learning disabilities and how we worked across four research projects with 867 participants (with one project still recruiting at the time of writing) to make online research more accessible to this group. We demonstrate how digital inclusion of people with learning disabilities is determined by a range of factors, including staff availability and willingness to support research engagement, access to technology, as well as researchers being flexible, adaptable, and ready to offer alternative or hybrid modes of participation. As more research moves to and stays online, we urge researchers to pay attention to how they design and conduct research so people with learning disabilities are not left behind in the process.

Digital exclusion has many dimensions and during the pandemic issues of access (and accessibility) intersected with digital skills and support (Coleman, 2021; Litchfield et al., 2021; Ramsetty & Adams, 2020). We know that the pandemic has had an impact on these dimensions, negatively affecting the well‐being of people with learning disabilities as well as those who support them (Lake et al., 2021; Sheerin et al., 2022), further limiting access and support (Navas et al., 2021) for many people. There is mixed evidence around the impact of the pandemic on the digital inclusion of people with learning disabilities. Some studies report increased inclusion; for example, a longitudinal study of aging in people with learning disabilities in Ireland found people reported increased family contact during the lockdown and increased the use of technology (McCausland et al., 2021). Others, like Chadwick and colleagues, observe that ‘COVID‐19 has not provided the impetus to eradicate digital poverty for people with intellectual disability’ (D. Chadwick et al., 2022; p. 1). We also know that self‐advocacy groups worked hard during the COVID‐19 pandemic to include and engage people online (People First Dorset, 2021).

There has been a historic lack of interest in the extent to which and how people with learning disabilities use technology (Seale et al., 2019). Pre‐pandemic studies provide some insights into the use of digital technologies and barriers to digital inclusion among people with learning disabilities. In a comparative study of the internet use of young people with and without learning disabilities in Sweden, Alfredsson Ågren et al. (2020a) found fewer people with learning disabilities accessed the internet, with information searching the greatest difference between the two groups. The importance of support and empowerment to enable effective internet access has also been highlighted (Molin et al. 2017; Shpigelman, 2018), particularly as people with learning disabilities might be at higher risk of online bullying (Alfredsson Ågren et al., 2020b). However, people can be further excluded if too much weight is given to perceived online risks—which might lead to gatekeeping and restrictions of access—but not enough effort is put into equipping people with skills to manage this risk and learn to navigate the online world (D. Chadwick, 2019). Other studies identify control over internet use by staff and/or families and safeguarding concerns, access to WIFI, and costs as barriers inhibiting digital engagement (Caton & Chapman, 2016; Alfredsson Ågren et al., 2020a; Martin et al., 2021). It has further been suggested that some researchers assume people with learning disabilities are not able to use technology (Seale et al., 2019). This is important and it matters for how research is designed and conducted.

Moreover, more people with learning disabilities are using devices such as smartphones to access the internet (Alfredsson Ågren et al., 2020a). This use has been associated with many benefits including social inclusion and relationships, leisure activities, the development of self‐esteem and enjoyment, with additional benefits of accessing information (Caton & Chapman, 2016; D. D. Chadwick et al., 2019; Martin et al., 2021; Seale et al., 2019; Shpigelman, 2018).

### 1.1 Moving research online

Doing research online presents challenges, including of ethical nature, throughout the research cycle (Lobe et al., 2020). Reduced opportunity to understand participants' context is one of the challenges identified, with online research also biased towards internet users (Pocock et al., 2021). More broadly, research suggests face‐to‐face interactions are more information‐rich than video‐mediated communication and we perceive people and situations differently online and in person (Davies et al., 2020; Croes et al., 2019; Basch et al., 2021; Schaarschmidt & Koehler, 2021). These points are important for online research, including for research on people with learning disabilities.

Although online research is not new, the rapid movement of nearly *all* research activities online to comply with government‐imposed curbs on in‐person interactions has the potential to fundamentally change the way research is conducted not just during the pandemic, but also beyond. Howlett (2022, p. 400) suggests ideological reasons in favour of digital methods:

the use of mediated methods can encourage greater collaboration and coordination between scholars [globally]… Further… as the pandemic continues, much more of our lives, and our participants', are being lived online, and thus, knowledge produced through physical immersion in a particular site may now be more ‘partial’ than ever before. Hence, the use of mediated methods not only challenges previously held understandings of the ‘field’… but has inspired new questions around conducting transparent, reflexive, and ethical research.

Such a possible long‐term shift is likely to have consequences for research with groups that continue to be digitally excluded. They risk being erased from potential research projects by their very design; for example, a study on how support for people with learning disabilities has been affected by the pandemic relied on an online survey and the authors observe:

given the need to administer the survey online because of the lockdown, people with IDD [intellectual and developmental disabilities] *that have difficulties accessing technology or have communication problems may have been unable to access the study, and have therefore been excluded* (Navas et al., 2021; p. 10; emphasis added).

The authors provide no details on whether they tried to address these issues. This is important as people with learning disabilities have been historically excluded from doing research and have been relegated to being research subjects (Lester & Nusbaum, 2018). However, even this relegation can be contingent as people with learning disabilities can be prevented from being the subjects of academic inquiry because the label of ‘learning disability’ can function as an exclusion criterion (Spaul et al., 2020). Within the field of learning disability research, there is an increasing, albeit still marginal, move towards more equal research practice that leads to more inclusive approaches for people with learning disabilities and other marginalised groups (Fletcher‐Watson et al., 2019; Mikulak et al., 2022; Nind, 2014; Warwick, 2020). Still, the question is whether historical exclusion from research paired with continuously high rates of digital exclusion for this group will result in new layers of marginalisation and if anything can be done to prevent it. Sharing insights on how digitalisation can support research with and by people with learning disabilities and highlighting the role of researchers in working to include people online we hope will highlight this issue and offer guidance on emerging practices in this area.

Finally, wider disability studies scholars have successfully used online and hybrid methods to coproduce research with disabled people (Liddiard et al., 2019). New technologies also offer new ways for people with learning disabilities to be included in research (Manning, 2010). As more research moves and potentially stays online, it is our duty as researchers to ensure that the meaningful inclusion of people with learning disabilities in research online also seeks to include those who might find themselves on the wrong side of the digital divide, challenging the latter in the process.

## 2 METHODS: RESEARCH DESIGN AND REDESIGN UNDER COVID‐19

This article presents our reflections on, and collective, and collaborative learning around doing qualitative research with and, for some of us, also *as* people with learning disabilities during the pandemic. We draw on four distinct research projects that we have worked on during the pandemic, with some of us involved in one of the projects, while others working across two, or three.

The process of developing and writing this paper happened in stages after the six Manchester Metropoliton University based academic authors (M.M., S. R., S. Ru., S. C., F. R., C. H.) met online to discuss our experiences of doing research during COVID‐19. We found similarities in the challenges and benefits of online, or hybrid models of doing research. We invited three of our collaborators with learning disabilities to contribute to a paper sharing insights on how digitalisation can support research with and by people with learning disabilities. Two (R. S. and R. K.‐B.) accepted the invitation. A provisional outline of the article based on our initial discussions was shared with academic coauthors by M. M. Contributions from R. K.‐B. and R. S. were sought separately, with S. C. and S. Ru. meeting with and recording their insights into doing/taking part in research online. Once all contributions were recorded on the shared document, M. M. and S. R. organised the paper into a draft, bringing together similarities and coalescing individual insights into shared accounts, while being attentive to differences in how the four projects, and different researchers, adapted and operated.

### 2.1 The projects

Project A, Coronavirus and People with Learning Disabilities, was a UK‐wide study that aimed to track the experiences of people with learning disabilities during the pandemic. A total of 692 people with learning disabilities were interviewed at three time points between December 2020 and August 2021. Structured interviews were conducted using Zoom, telephone, WhatsApp, Microsoft Teams or Facetime (according to participant preference). Multiple recruitment methods included collaborating organisations in each UK country, social media and wider networks of learning disability and family organizations. Flexibility was key to ensuring that people could participate in their preferred way. Interviews typically lasted 45 minutes and were usually completed in one sitting with a short break as needed. At each wave of interviews, people with learning disabilities across the four UK countries were consulted about the interview questions to maximise relevance and accessibility. Project A was the only project of the four designed and funded during the pandemic and there was little need for adaptation. The only unexpected change was the adoption of Zoom which became the platform of choice for most participants.

Project B, Flourishing Lives, aimed to explore what ‘good’ social care and support for people with learning disabilities look like and how it can be delivered in practice. Components of good care, generated from a scoping literature review, were used to guide discussion with participants in online focus groups and interviews. Fieldwork took place between October 2020 and March 2021. Interviews were offered as an alternative to focus groups which can be challenging for people with learning disabilities (Kaehne & O'Connell, 2010). Recruitment was via self‐advocacy groups and charities, project advisory group members' networks, and social media. A total of 20 interviews and 14 focus groups were conducted with 50 people with learning disabilities and 28 family carers. Interviews lasted up to an hour and focus groups were around 90 minutes. Where possible the researchers met online with participants in advance to explain the project, answer any questions and resolve technical issues. More and smaller focus groups than originally planned were held to adapt to the online format. Microsoft Teams was the prescribed platform for fieldwork by the university.

Project C, Growing Older Planning Ahead, aims to improve support for family and professional carers and older people with learning disabilities. Part of the study included identifying exemplars of good practice in services and support interventions in the United Kingdom for older people with learning disabilities and their family and professional carers, and exploring these service exemplars through ethnographic research. A four to eight‐site ethnography involves observations, interviews and documentary analysis of existing policies with participants recruited through support providers. The sites were chosen to demonstrate a diversity of approaches to provision, with four types of support identified: independent supported living; residential services; family/home‐based support and daytime activities and services; and Shared Lives (NHS, 2021). Each site includes 20 ethnography days and interviews with people with learning disabilities, family carers, support workers, relevant commissioners and service managers. In total around 100–115 interviews will be conducted across the four to eight sites. Ethnographic coresearchers (people with learning disabilities and family carers) are accompanying academic researchers on up to 50% of the ethnographic visits. A hybrid approach was adopted with ethnographic work taking place when it was possible and safe to do so. The time during heightened restrictions was used for interviews and to meet participating people with learning disabilities informally online, for short 15–30 minute Zoom meetings (3–5 times). These meetings involved chatting about everyday life and getting to know each other. This did not work for all participants as some did not communicate with words or have enough support to facilitate online participation.

Project D, 200 Lives, aimed to examine the quality and costs of supported living and residential care for adults with learning disabilities in England. It was designed to seek the perspectives of up to 200 adults with learning disabilities aged between 18 and 74 living in residential care or supported living including people who did not have the capacity to take part in the research. Fieldwork took place between March and December 2021. A combination of interviews and questionnaires included interviews with adults with learning disabilities, a questionnaire for support staff, a household questionnaire completed by support staff, a survey completed by a close family member, and Quality of Life reviews. Here we focus on the interviews. Recruitment was via support providers. A flexible approach was taken to reflect evolving COVID‐19 restrictions and ensure participants were comfortable and individual preferences respected. While most interviews took place using telephone, Zoom, Teams or WhatsApp, some were face‐to‐face, and a small number of participants completed the interview with their support provider and sent it back to the research team by post. Interviews were typically split into two sessions of around 60 minutes each though some participants preferred multiple shorter sessions across several weeks. A total of 107 participants from 16 providers took part (93 participants with the capacity to consent and 14 for whom proxy consent was collected) in approximately 215 interviews.

## 3 FINDINGS AND DISCUSSION: SILVER LININGS AND NEW WAYS OF WORKING TOGETHER

In this section, we discuss how moving and doing research online gave us an opportunity to develop different, often more accessible ways of working with people with learning disabilities as research participants, members of advisory groups and coresearchers. We begin by sharing insights from research team members with learning disabilities. While many of the issues raised in this first section map onto and inform our wider discussion, we highlight these insights separately because it is important to not subsume and potentially make invisible contributions from people with learning disabilities in coauthored papers (Hopkins et al., 2022).

Author R. K.‐B. was a research participant and member of the virtual advisory group for Project A and a researcher on Project C. He shared how he valued new professional and personal connections created by meetings during lockdowns while there were also disadvantages: ‘you miss out on seeing people face‐to‐face with only seeing them on a screen. It's just nice if you are working with someone to meet them properly for a personal connection’.

R. K.‐B. observed that because members of the advisory group were first invited to join the group through email contact, they needed existing digital skills to get involved; a point we revisit below. He said:

Internet is easy if you know how to use it, but some people will find it a problem. If someone hadn't set up Zoom and Teams for me, I wouldn't have been able to use it and it wouldn't have been so easy to use it and we'd have had to use the telephone.

At the same time, R. K.‐B. thought taking part in the study improved digital skills and allows participants:

to feel relaxed in their own home, with no need to travel anywhere, and enjoying home comforts while taking part, you don't have to travel too far. You can sit in your armchair in your slippers and turn on your computer.

He further reflected; ‘It's a lot of effort to get ready to go out, so it's a lot easier to just turn on your computer’ (R. K. B.). These pragmatic benefits are echoed in our reflections across all projects.

Being able to see the researcher's background can be a topic for an ‘ice‐breaking’ initial conversation: ‘it's nice to see what people have got in their rooms – it's a good way to make conversation with people’ (R. K.‐B.). We return to this point when we discuss how serendipitous interactions enriched our data and how there is a new sense of reciprocity to online interactions.

R. K.‐B.'s final reflection was about how Project A would not have been able to involve so many people in the research without the use of online methods ‘because there were people from Yorkshire, London, Manchester, and all sorts of parts of the country’.

It was the first time Author R. S., a member of Project B's advisory group, had taken part in the research. Supported by her father, she was comfortable with the use of online platforms, with a preference for Zoom, and described how she liked the combination of a large group and smaller break‐out rooms during project meetings:

I like it when we talk in one big group so I can hear what people think and I like the small breakout rooms groups where I feel more confident asking questions. I like it when we all get together as a team and talk about how the project is going and the things we have found out, including about how important it is that we're all part of the community (RS).

The key issue for R. S. was allowing space and time during online meetings for her to speak:

*‘*Sometimes in groups people just think that I'm not going to say anything and don't give me a chance to get started talking’. She also felt it is easier for people to be talked over or ignored during online meetings. We return to the importance of planning and offering alternative ways for people to engage so their contributions are not lost below.

### 3.1 Pragmatic responses and benefits

All four projects experienced similar benefits of conducting the research online and saving time and money related to fieldwork travel for researchers; holding online advisory or coresearcher meetings facilitates attendance and cuts down on the time commitment for everyone involved. Furthermore, the absence of researcher travel costs and time removed the pressure on researchers to complete interviews in one sitting, allowing them to better respond to participants' individual preferences, for example, adapting the length of sessions to suit how participants felt on the day. A key benefit to moving research online was greater flexibility in how researchers work with people with learning disabilities as participants, opening space for innovation and in effect making research more accessible.

The new circumstances demanded pragmatic responses to enable the projects to continue. For example, recruitment of participants for Project D was delayed and as the pandemic developed, some providers who expressed interest in the project felt unable to participate due to limited staffing capacity. When providers took part, they tended to put forward fewer participants than originally anticipated. To increase participant numbers, project D broadened some eligibility criteria and (with additional funding) extended the data collection time.

The often‐practical adaptations by research teams delivered unexpected benefits and generated new insights about how research can be done better beyond the pandemic. In Project C, not all participants could take part in informal Zoom meetings between ethnography visits, which created a de facto control group to evaluate how helpful these initial meetings were for building rapport and getting to know the participants, their support team, and learning about their lives. We found these introductory meetings were valuable for the researcher and participants seemed to enjoy them. They built familiarity and excitement for the in‐person visits, as participants would discuss the things and places they wanted to show the researcher when they met offline. The meetings continued between ethnography visits allowing for continuity of engagement and providing space for follow‐up questions arising from visits, and/or new developments in participants' lives. The meetings added a new and unexpected richness to the longitudinal aspects of ethnography which would not have been achieved with face‐to‐face research visits only.

### 3.2 Broadening collaborations and exploring alternative ways of doing things

The coresearch training on Project C had to be conducted online. This created a new set of demands on the research team but also enabled us to coproduce a set of training resources with a geographically spread team of collaborators. Similarly, the training which was delivered to nine coresearchers (eight people with learning disabilities and one family carer) was attended by coresearchers from Greater Manchester, Dorset and Oxfordshire allowing us to build crucial capacity local to our research sites. When the coresearch took place, it was with a coresearcher with extensive knowledge of the local area, often able to fill in the gaps and provide context and information that the research team did not have.

At times, being adaptable meant creating alternatives to activities that were difficult to transfer online. On Project B, the original design included an ordering exercise for small group work which involved physically sorting through items from the component list. This was challenging to recreate online, and the list was shared as easy‐read items on a PowerPoint slide. Participants were invited to direct the researcher to move items around the slide depending on the relevance or importance of each. In other instances, being adaptable meant giving something up altogether. On Project D, a series of easy‐read flashcards had been developed to be used alongside the interview questions to aid understanding of the questions being asked. Despite adapting the flashcards for online use, they did not work well across the different platforms. The researchers struggled to focus on the participants and respond to nonverbal cues while sharing their screen and found it difficult to scroll to the relevant flashcard when the conversation moved, or questions were asked out of order. Eventually, the flashcards were discarded, and the project would have benefited from exploring alternative supportive tools. These experiences highlight the need for innovation in our online research practice, as we look for effective tools and methods.

As noticed above by R. K.‐B., doing research online can be more comfortable for both the researchers and participants. For Project A, participants were asked to take part in three interviews, and we found a low attrition rate between interviews, perhaps because participants enjoyed the social interaction at a time when lives were more confined. On Projects A and C, we also experienced additional benefits of participants typically being at home as it enabled serendipitous interactions that enriched the data. This included sharing artwork, giving the researcher a virtual tour of the house or garden, or simply showing what the weather was like on that day. As notable also on Project C, online meetings created a new sense of reciprocity between researchers and participants, as the latter gained glimpses of researchers' homes, lives, plants and pets. The human connection and fun embedded in these interactions deserve to be acknowledged and celebrated as new.

### 3.3 Small adaptations, big difference

There are additional steps and work that might be required to engage people in research online. For Projects B and C, and for the first wave of Project A, considerable behind‐the‐scenes work was conducted to support people to attend, and the projects benefited from exemplary support from self‐advocacy groups who took responsibility to ensure people could join the groups and remained present to support their participation. On Project C, while some coresearchers were supported by self‐advocacy organizations, others relied on the research team for support. To ensure attendance, researchers phoned and/or texted coresearchers with information about the training as well as emailing it and sending it out in the post (with attention paid to large font and paper colour preferences). Posting printed presentation slides beforehand gave coresearchers an opportunity to prepare for the training. In Project A, researchers emailed participants links to join calls and sent a reminder email with the link on the day of the interview. These seemingly simple adaptations and practices can make a difference to someone's ability and confidence around taking part and they should be developed early and folded into research designs.

### 3.4 More accessible, but still not for all

To make online research accessible, researchers had to push back against arbitrary rules about software to respond to the preferences of participants and collaborators (e.g. author's, R. S.). On Project B, the initial insistence by the university on the use of Microsoft Teams caused issues for participants who were familiar with and preferred Zoom. After pressure from the research team, the project was granted exceptional permission to use Zoom. On Project C, a special request had to be made for a Zoom license, as coresearchers were unanimous in their preference for it.

Finally, designing and keeping research accessible means acknowledging that taking part digitally is not an option for some participants and offering offline alternatives. On Project D, a flexible approach to data collection was taken to allow people to take part in ways that suited them. For example, some people opted to delay participation until they were able to meet in person, while a small number of participants took part by answering the interview questions with their support staff rather than online with a researcher.

### 3.5 New ways of doing things, same old (and some new) challenges

Online research poses challenges that researchers encounter also when working with people with learning disabilities offline. However, some of these challenges can be magnified by online interactions. Most notably, all four projects encountered issues surrounding gatekeeping and an increased reliance on staff and staff availability to facilitate fieldwork and meetings. Some people who would have liked to participate were unable to do so. The necessity of arranging recruitment and interviews through a gatekeeper meant that if that person was unavailable (staff absences were common as people were frequently required to self‐isolate) the interview could not go ahead in Projects A and D. This was also an issue for Project C, where short Zoom meetings with participants would often be rescheduled or cancelled last minute because of a lack of staff. Due to the pandemic, some providers felt unable to support participation. In Project C, it took one provider almost a year to join the study, with several delays and cancellations happening along the way.

In Project A, there were difficulties whereby gatekeepers and interviewers attempted to arrange interview times with a pragmatic time‐saving approach, grouping interview times together (e.g., using Zoom breakout rooms or where a support worker could support a few participants in succession, in‐person). If difficulties arose on the day meaning a participant could not attend, it often proved difficult to rearrange interviews.

### 3.6 Gatekeeping and digital exclusion

Moreover, while the digital gap might be narrowing for people with learning disabilities, for many access to technology remains mediated through family and support workers. This was true for many of our participants who were reliant on support staff to facilitate access to technology and/or a particular platform. Some participants did not have their own devices and needed a staff member with access to a laptop to be on shift. Others had their own device but were not comfortable using it independently, especially when using an unfamiliar platform such as Zoom.

These issues might *appear* new and were amplified by the uncertainties of the pandemic reality, but they also point to a key question that is older than the internet. Namely, how do we as researchers make the point and importance of our research better understood? How do we communicate the aims of our work in ways that generate interest and engagement, thereby facilitating a more effective relationship with gatekeepers?

Gatekeeping, combined with digital inclusion and skills gaps meant interviews or meetings were often missed, or cancelled at short notice. At the same time, little effort was put into supporting people to acquire skills that would allow them to take part independently. It poses a further question of how much weight is given to developing and maintaining digital skills that might lessen the pressure on support staff and give people with learning disabilities more control and agency to take part in research, but also to access the known benefits of being online more broadly.

### 3.7 Privacy concerns and risk of erasure

Online research can pose challenges around confidentiality and privacy in ways that can be difficult to control by researchers. For Projects B and C, it was not always possible to ensure participants had privacy to take part in fieldwork and there could be interruptions from other household members or staff. For Project B, the lack of privacy related to taking part in a group activity was mitigated to some extent by the mute function, and it was apparent that people were becoming more adept at managing their involvement in online meetings. This echoes R. B. K.'s comment about improved digital literacy as a result of taking part in research. For Projects C and D, it was not always possible to know who else was in the room with the participant, or who else might be listening. This requires researchers to be attentive to what is beyond the screen and how that might affect the participant and the data.

Finally, doing research online risks reproducing inequalities that people with learning disabilities face in research and in society at large. Project A was designed with recruitment via digital means including electronically distributed newsletters from collaborating organizations, email, self‐advocacy groups, and social media. People with learning disabilities were usually invited to participate through gatekeepers from supported living or residential care but people living independently who were not regular internet users were unlikely to have received information about the study. Therefore, it is likely that the main barrier to participation was that many people would not have known about the study. This has ethical and empirical implications and points to the need to consider whose voices will be erased in online research and whose responsibility it is to prevent this erasure from happening.

## 4 CONCLUSION

COVID‐19 has shown that research can adapt extremely quickly to new circumstances and challenges. While many of these adaptations, including the move online, can make it more accessible for some people with learning disabilities, there are risks associated with further excluding those for whom it is not possible to engage online. People with learning disabilities continue to face digital exclusion and the importance of support and empowerment flagged up in the prepandemic literature is reinforced by our projects. In some ways, the pandemic has fast‐tracked this process as providers, self‐advocacy groups and families worked to ensure that (some) people had the knowledge, skills and devices to interact online during lockdowns. However, consideration is urgently needed around what digital inclusion in research would mean for some groups of people, such as people with profound and multiple learning disabilities, who are typically excluded from research. We suggest further coproduced research is needed to widen the range of online research methods, particularly creative approaches that are more inclusive for all people with learning disabilities.

Across the four projects, we show that taking part in research has the potential to improve digital inclusion and skills, especially for research‐based on coproduction and long‐term engagement with people with learning disabilities as participants and partners. These benefits should be harnessed and further developed in the postpandemic world, as we continue to work and innovate to make research more inclusive and thus better.

We also demonstrate how research practices are improved by the additional reflection that online participation generates and by the flexibility digital technologies can offer. For example, it offers opportunities to meet with advisory panel members outside of formal meetings to discuss emerging issues, facilitates breakout meetings, makes it possible to get to know participants ahead of fieldwork, and facilitates local capacity building for coresearch. Online research also benefits from serendipitous interactions and insights that can further enrich the data and knowledge produced.

We have discussed how being flexible and adaptable can allow more people with learning disabilities to take part in research. Such flexibility means more than just offering a choice between Zoom and Microsoft Teams; hybrid approaches that combine face‐to‐face with online methods might work better, to ensure people who are digitally excluded are not left out of recruitment and participation. This is key and we argue that it should no longer be acceptable to simply state that people who are unable to participate online have been excluded from any given research. Active investigation of barriers to participation and genuine efforts to overcome these should be the new standard, and researchers need to respond to this challenge with commitment, integrity, and creativity. Research can and should adapt to challenge exclusion and it is our responsibility to ensure that the way we work does not reproduce the inequalities our research sets out to investigate, critique and transform.

Furthermore, while this paper foregrounds our reflections and insights as *researchers* and collaborators on research projects, to better understand the consequences of the move to online research, future studies should explore the experiences and perspectives of people with learning disabilities as research *participants*.

Research participation is at times only possible through collaboration with support staff and family carers. Researchers should continue to advocate for and promote the right of people with learning disabilities to participate in research, independently or with support, even at times of added difficulty—such as a pandemic—to ensure the voices and participation of people who are already facing digital exclusion are not erased further. We recommend that researchers:

- Plan which elements of the research will be online, with a clear rationale and assessment of who is likely to be excluded;
- Build flexibility into the research design to remain adaptive;
- Consider hybrid approaches, combining online and face‐to‐face methods and alternatives;
- Ensure institutional support for the online platforms required for the research to be accessible and consistent with the preferences of coresearchers, advisory group members, and participants;
- Use online methods to build and maintain working relationships with people, as coresearchers and/or as participants;
- Allocate time and resources to work with ‘gatekeepers’ and facilitators to secure wider participation;
- Consider issues of privacy when conducting online research;
- Make coproduction central to ensure that our methods are accessible and effective.

## ACKNOWLEDGEMENTS

The Coronavirus and People with Learning Disabilities Study was funded by UK Research and Innovation (Medical Research Council) and supported by the Department for Health and Social Care (National Institute for Health Research) as part of the UKRI‐DHSC COVID‐19 Rapid Response Rolling Call. UKRI: MR/V028596/1 NIHR: COV0196. The views expressed in this publication are those of the authors and not necessarily those of DHSC, NIHR, UKRI, or MRC. The Flourishing Lives project is funded by National Institute for Health Research School for Social Care Research. This paper is independent research by the National Institute for Health Research School for Social Care Research. The views expressed in this publication are those of the author(s) and not necessarily those of the NIHR SSCR, the National Institute for Health Research or the Department of Health and Social Care. The Growing Older Planning Ahead research is funded by the National Institute for Health Research (NIHR) under its Health Services and Delivery Research Programme (NIHR129491). The views expressed are those of the authors, and not necessarily those of the NHS, the NIHR or the Department of Health and Social Care. The 200 Lives project is funded by the National Institute for Health Research (NIHR) under its Research for Patient Benefit (RfPB) Programme (Grant Reference Number NIHR200069). The views expressed are those of the authors and not necessarily those of the NIHR or the Department of Health and Social Care.

## CONFLICT OF INTEREST

The authors declare no conflict of interest.

## References

[bld12495-bib-0001] Alfredsson Ågren, K. , Kjellberg, A. , & Hemmingsson, H. (2020a). Digital participation? Internet use among adolescents with and without intellectual disabilities: A comparative study. New Media & Society, 22(12), 2128–2145.

[bld12495-bib-0002] Alfredsson Ågren, K. , Kjellberg, A. , & Hemmingsson, H. (2020b). Internet opportunities and risks for adolescents with intellectual disabilities: A comparative study of parents' perceptions. Scandinavian Journal of Occupational Therapy, 27(8), 601–613. 32538241 10.1080/11038128.2020.1770330

[bld12495-bib-0003] Basch, J. M. , Melchers, K. G. , Kurz, A. , Krieger, M. , & Miller, L. (2021). It takes more than a good camera: Which factors contribute to differences between face‐to‐face interviews and videoconference interviews regarding performance ratings and interviewee perceptions? Journal of Business and Psychology, 36(5), 921–940. 32929301 10.1007/s10869-020-09714-3 PMC7482058

[bld12495-bib-0045] Caton, S. , & Chapman, M . (2016). The use of social media and people with intellectual disability: A systematic review and thematic analysis. Journal of Intellectual & Developmental Disability, 41(2), 125–139. 10.3109/13668250.2016.1153052

[bld12495-bib-0005] Chadwick, D. (2019). Online risk for people with intellectual disabilities. Tizard Learning Disability Review, 24(4), 180–187.

[bld12495-bib-0006] Chadwick, D. , Ågren, K. A. , Caton, S. , Chiner, E. , Danker, J. , Gómez‐Puerta, M. , Heitplatz, V. , Johansson, S. , Normand, C. L. , Murphy, E. , Plichta, P. , Strnadová, I. , & Wallén, E. F. (2022). Digital inclusion and participation of people with intellectual disabilities during COVID‐19: A rapid review and international bricolage. Journal of Policy and Practice in intellectual Disabilities , 1– 15.

[bld12495-bib-0007] Chadwick, D. D. , Chapman, M. , & Caton, S. (2019). Digital inclusion for people with an intellectual disability. In A. Attrill‐Smith , C. Fullwood , M. Keep , & D. J. Kuss (Eds.), The Oxford handbook of cyberpsychology. Oxford University Press.

[bld12495-bib-0008] Coleman, V. (2021). Digital divide in UK education during COVID‐19 pandemic: Literature review. Retrieved January 28, 2022, from https://files.eric.ed.gov/fulltext/ED616296.pdf

[bld12495-bib-0046] Croes, E. A. J. , Antheunis, M. L. , Schouten, A. P. , & Krahmer, E. J . (2019). Social attraction in video‐mediated communication: The role of nonverbal affiliative behavior. Journal of Social and Personal Relationships, 36(4), 1210–1232. 10.1177/0265407518757382 30886451 PMC6394910

[bld12495-bib-0009] Davies, L. , LeClair, K. L. , Bagley, P. , Blunt, H. , Hinton, L. , Ryan, S. , & Ziebland, S. (2020). Face‐to‐Face compared with online collected accounts of health and illness experiences: A scoping review. Qualitative Health Research, 30(13), 2092–2102. 32667257 10.1177/1049732320935835

[bld12495-bib-0010] Fletcher‐Watson, S. , Adams, J. , Brook, K. , Charman, T. , Crane, L. , Cusack, J. , Leekam, S. , Milton, D. , Parr, J. R. , & Pellicano, E. (2019). Making the future together: Shaping autism research through meaningful participation. Autism: The International Journal of Research and Practice, 23(4), 943–953. 30095277 10.1177/1362361318786721 PMC6512245

[bld12495-bib-0013] Hantrais, L. , Allin, P. , Kritikos, M. , Sogomonjan, M. , Anand, P. B. , Livingstone, S. , Williams, M. , & Innes, M. (2021). Covid‐19 and the digital revolution. Contemporary Social Science, 16(2), 256–270.

[bld12495-bib-0014] Hopkins, R. , McGrath, J. , Hogan, B. , Skehan, P. , & Acheson, L. (2022). In response to ‘Ethno…graphy?!? I can't even say it’: Co‐designing training for ethnographic research for people with learning disabilities and carers. British Journal of Learning Disabilities, 50, 61–65.

[bld12495-bib-0015] Howlett, M. (2022). Looking at the ‘field’ through a zoom lens: Methodological reflections on conducting online research during a global pandemic. Qualitative Research, 22(3), 387–402. 35663097 10.1177/1468794120985691 PMC9095994

[bld12495-bib-0016] Kaehne, A. , & O'Connell, C. (2010). Focus groups with people with learning disabilities. Journal of Intellectual Disabilities, 14(2), 133–145. 20930023 10.1177/1744629510381939

[bld12495-bib-0017] Knights, F. , Carter, J. , Deal, A. , Crawshaw, A. F. , Hayward, S. E. , Jones, L. , & Hargreaves, S. (2021). Impact of COVID‐19 on migrants' access to primary care and implications for vaccine roll‐out: A national qualitative study. British Journal of General Practice, 71(709), e583–e595. 10.3399/BJGP.2021.0028 PMC8216266 33875420

[bld12495-bib-0018] Lake, J. K. , Jachyra, P. , Volpe, T. , Lunsky, Y. , Magnacca, C. , Marcinkiewicz, A. , & Hamdani, Y. (2021). The wellbeing and mental health care experiences of adults with intellectual and developmental disabilities during COVID‐19. Journal of Mental Health Research in Intellectual Disabilities, 14(3), 285–300.

[bld12495-bib-0019] Lester, J. N. , & Nusbaum, E. A. (2018). “Reclaiming” disability in critical qualitative research: Introduction to the special issue. Qualitative Inquiry, 24(1), 3–7.

[bld12495-bib-0020] Liddiard, K. , Runswick‐Cole, K. , Goodley, D. , Whitney, S. , Vogelmann, E. , & Watts MBE, L. (2019). “I was excited by the idea of a project that focuses on those unasked questions” co‐producing disability research with disabled young people. Children & Society, 33(2), 154–167.

[bld12495-bib-0044] Litchfield, I. , Shukla, D. , & Greenfield, S . (2021). Impact of COVID‐19 on the digital divide: A rapid review. *BMJ open* , 11(10), e053440. 10.1136/bmjopen-2021-053440 PMC8520586 34642200

[bld12495-bib-0021] Lobe, B. , Morgan, D. , & Hoffman, K. A. (2020). Qualitative data collection in an era of social distancing. International Journal of Qualitative Methods, 19, 160940692093787. 10.1177/1609406920937875

[bld12495-bib-0022] Manning, C. (2010). ‘My memory's back!’ inclusive learning disability research using ethics, oral history and digital storytelling. British Journal of Learning Disabilities, 38(3), 160–167.

[bld12495-bib-0023] Martin, A. J. , Strnadová, I. , Loblinzk, J. , Danker, J. C. , & Cumming, T. M. (2021). The role of mobile technology in promoting social inclusion among adults with intellectual disabilities. Journal of Applied Research in Intellectual Disabilities, 34(3), 840–851. 33675142 10.1111/jar.12869

[bld12495-bib-0024] McCausland, D. , Luus, R. , McCallion, P. , Murphy, E. , & McCarron, M. (2021). The impact of COVID‐19 on the social inclusion of older adults with an intellectual disability during the first wave of the pandemic in Ireland. Journal of Intellectual Disability Research, 65(10), 879–889. 34165228 10.1111/jir.12862 PMC8447302

[bld12495-bib-0025] Mikulak, M. , Ryan, S. , Bebbington, P. , Bennett, S. , Carter, J. , Davidson, L. , Liddell, K. , Vaid, A. , & Albury, C. (2022). “Ethno…graphy?!? I can't even say it”: Co‐designing training for ethnographic research for people with learning disabilities and carers. British Journal of Learning Disabilities, 50(1), 52–60.

[bld12495-bib-0026] Molin, M. , Sorbring, E. , & Löfgren‐Mårtenson, L. (2017). New em@ ncipatory landscapes? Young people with intellectual disabilities, internet use, and identification processes. Advances in Social Work, 18(2), 645–662.

[bld12495-bib-0027] Navas, P. , Amor, A. M. , Crespo, M. , Wolowiec, Z. , & Verdugo, M. Á. (2021). Supports for people with intellectual and developmental disabilities during the COVID‐19 pandemic from their own perspective. Research in Developmental Disabilities, 108, 103813. 33271448 10.1016/j.ridd.2020.103813 PMC7651232

[bld12495-bib-0028] NHS . (2021). Shared lives schemes. Retrieved January 28, 2022, from https://www.nhs.uk/conditions/social-care-and-support-guide/care-services-equipment-and-care-homes/shared-lives-schemes/

[bld12495-bib-0029] Nind, M. (2014). What is inclusive research? (1 ed.). Bloomsbury Academic.

[bld12495-bib-0030] Office for National Statistics . (2019). Exploring the UK's digital divide. Retrieved January 28, 2022, from file:///C:/Users/55141817/Downloads/Exploring%20the%20UK%20s%20digital%20divide.pdf

[bld12495-bib-0032] Peckham, E. , & Spanakis, P. (2020). Online or on the line? Digital exclusion in people with severe mental ill health. Retrieved January 28, 2022, from https://mentalhealthresearchmatters.org.uk/online-or-on-the-line-digital-exclusion-in-people-with-severe-mental-ill-health/

[bld12495-bib-0033] People First Dorset . (2021). Covid‐19 impact report. Retrieved January 28, 2022, from https://www.peoplefirstdorset.org.uk/_files/ugd/d7994b_e23a770040a34cbfb4b4a11e827507ad.pdf

[bld12495-bib-0034] Pocock, T. , Smith, M. , & Wiles, J. (2021). Recommendations for virtual qualitative health research during a pandemic. Qualitative Health Research, 31(13), 2403–2413. 34384307 10.1177/10497323211036891

[bld12495-bib-0035] Ramsetty, A. , & Adams, C. (2020). Impact of the digital divide in the age of COVID‐19. Journal of the American Medical Informatics Association, 27(7), 1147–1148. 32343813 10.1093/jamia/ocaa078 PMC7197532

[bld12495-bib-0047] Schaarschmidt, N. , & Koehler, T . (2021). Experiencing emotions in video‐mediated psychological counselling versus to face‐to‐face settings. Societies, 11(1), 1–10. https://EconPapers.repec.org/RePEc:gam:jsoctx:v:11:y:2021:i:1:p:20-:d:515276

[bld12495-bib-0037] Seale, J. , Choksi, A. , & Spencer, K. (2019). ‘I've been a whizz‐kid since I've been at college’: Giving voice to the collective memories of adults with learning disabilities about the role that technology has played in their lives. Disability Studies Quarterly, 39(4), 6621–551.

[bld12495-bib-0038] Seifert, A. (2020). The digital exclusion of older adults during the COVID‐19 pandemic. Journal of Gerontological Social Work, 63(6–7), 674–676. 32401181 10.1080/01634372.2020.1764687

[bld12495-bib-0039] Sheerin, F. , Allen, A. P. , Fallon, M. , McCallion, P. , McCarron, M. , Mulryan, N. , & Chen, Y. (2022). Staff mental health while providing care to people with intellectual disability during the COVID‐19 pandemic. British Journal of Learning Disabilities , 1–11. 10.1111/bld.12458 PMC9111622 35602324

[bld12495-bib-0040] Shpigelman, C. N. (2018). Leveraging social capital of individuals with intellectual disabilities through participation on facebook. Journal of Applied Research in Intellectual Disabilities, 31(1), e79–e91. 28000333 10.1111/jar.12321

[bld12495-bib-0041] Spaul, S. W. , Hudson, R. , Harvey, C. , Macdonald, H. , & Perez, J. (2020). Exclusion criterion: Learning disability. The Lancet, 395(10223), e29. 10.1016/S0140-6736(20)30051-9 32061300

[bld12495-bib-0042] Warwick, M. (2020). Shifting the gaze: The use of wearable cameras in research. In M. Nind & I, Strnadová (Eds.), Belonging for people with profound intellectual and multiple disabilities: Pushing the boundaries of inclusion (pp. 113–126).

